# A Discrete Hemodynamic Control System: Proof-of-Concept In-Vivo Study in Canine Heart Failure Using a Pulmonary Artery Catheter

**DOI:** 10.1109/OJEMB.2026.3723319

**Published:** 2026-08-12

**Authors:** Yasuyuki Kataoka, Kazunori Uemura, Takuya Nishikawa, Yukiko Fukuda, Jon Peterson, Kei Sato, Hiroki Matsushita, Hidetaka Morita, Yuta Nakamura, Masahiro Otake, Kenta Ohba, Nana Hiraki, Keita Saku, Mitsuji Sampei, Joe Alexander, Kenji Sunagawa

**Affiliations:** ^1^ NTT Research, Inc. Sunnyvale CA 94085 USA; ^2^ National Cerebral and Cardiovascular Center 13875 Suita 564-8565 Japan; ^3^ Institute of Science Tokyo 13290 Meguro-ku 152-8550 Japan; ^4^ Circulatory System Research Foundation Bunkyo-ku 113-0033 Japan

**Keywords:** Hemodynamic control system, in-vivo validation, heart failure

## Abstract

*Objective:* Managing heart failure requires simultaneous regulation of mean arterial pressure, cardiac output, and left atrial pressure, which is challenging due to complex cardiovascular interactions and limited monitoring. This study presents a proof-of-concept in-vivo evaluation of a discrete two-degree-of-freedom hemodynamic control system designed to regulate these three hemodynamic variables through titration of cardiovascular interventions under pulmonary artery catheter monitoring. *Results:* Across five consecutive trials in three canine models of heart failure induced by tachycardia pacing, the cohort-averaged mean arterial pressure, cardiac output, and left atrial pressure were each within *±*10% of their respective targets when evaluated using the final three intervention measurements, with absolute percentage errors decreasing from 16.8% to 9.0%, 23.4% to 3.8%, and 87.5% to 5.5%, respectively. In a more severely complicated heart failure model with coronary embolization, outcomes revealed control limitation primarily due to pharmacological resistance, highlighting the importance of choosing the appropriate target value. *Conclusions:* This proof-of-concept in-vivo study demonstrates the feasibility of implementing discrete two-degree-of-freedom hemodynamic control under pulmonary artery catheter-based monitoring and phlebotomy-mediated volume removal in anesthetized canine models of tachycardia-pacing-induced heart failure. Further studies are required to evaluate the effects of inter-subject variability and clinical generalizability.

## I. Introduction

### A. Clinical Challenges in Hemodynamic Management

In managing patients with heart failure (HF), major guidelines recommend optimizing mean arterial pressure (*MAP*), cardiac output (*CO*), and left atrial pressure ($P_{LA}$) to preserve organ perfusion and reduce pulmonary congestion [1], [2], [3]. Simultaneous control of *MAP*, *CO*, and $P_{LA}$ remains difficult even for experienced clinicians due to complex cardiovascular interactions and limited monitoring.

Autonomous pharmacologic control systems of the three hemodynamic variables have been proposed, yet many depend on continuous measurements of the metrics through highly invasive sensors under thoracotomy, restricting their clinical applicability [4], [5], [6].

In intensive care or coronary care units (ICUs or CCUs) using a pulmonary artery catheter (PAC), *CO* and $P_{LA}$ are usually measured intermittently by thermodilution technique and catheter balloon inflation, which reflects the pulmonary artery wedge pressure (*PAWP*), respectively [7], [8]. Noninvasive continuous *CO* monitoring methods exist, yet their accuracy remains limited [9].

### B. Hemodynamnic Control System

Since *CO* and $P_{LA}$ are discretely available in real clinical practice, we developed a discrete two-degree-of-freedom (2DoF) hemodynamic control framework [10], consisting of feedforward and feedback control. Unlike previous approaches relying on continuous hemodynamic monitoring under thoracotomy, the proposed framework is designed for PAC-based discrete measurements routinely available in clinical practice. Fig. 1 shows one implementation, where the input $\bm {u}$ comprises five interventions, the state $\bm {x}$ includes six cardiovascular parameters, and the output $\bm {y}$ denotes five hemodynamic variables. A discrete step spans from a drug infusion update to hemodynamic stabilization. At each step, $\bm {u}$ is updated once. Over the multiple steps, $\bm {y}_{1:3}$ are regulated. Previous in-silico studies validated the system [10] (supplementary materials S-I & S-II). Translating it in vivo, however, raises critical concerns.

**Figure 1.**
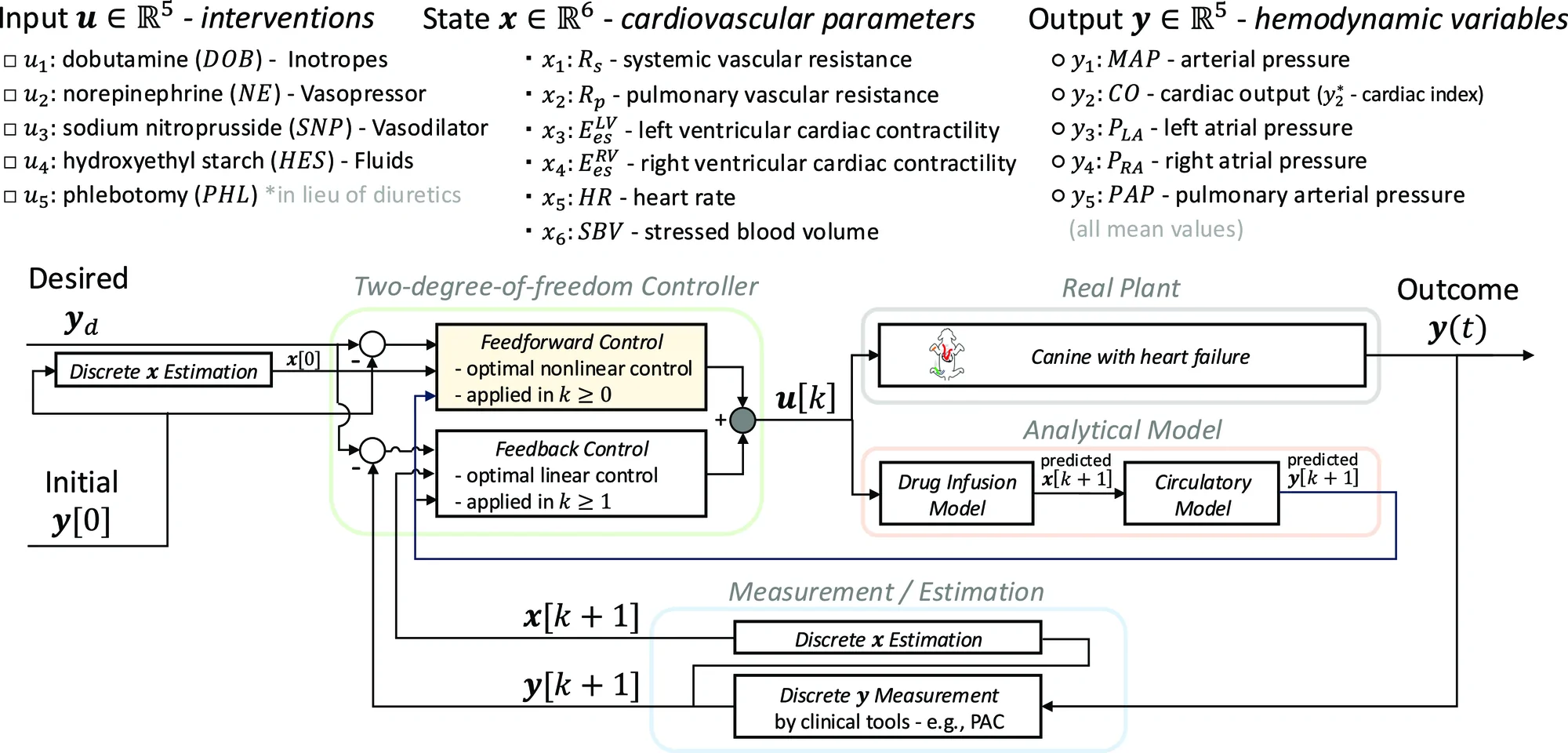
Overview of Discrete Two-Degree-of-Freedom Hemodynamic Control System.

First, measurement and estimation errors can affect control performance. Second, unavoidable discrepancies between the analytical model used in the system (Fig. 1) and the actual in-vivo physiology can also affect performance. The analytical model is grounded in physiological causality using a drug infusion model ($\bm {u} \rightarrow \bm {x}$) and circulatory model ($\bm {x} \rightarrow \bm {y}$) [10], [11]. The discrepancies may arise from model simplifications and parameter uncertainties, including variability in drug sensitivities.

To date, this system has not been tested in vivo for its ability to suppress these disturbances.

### C. Study Objectives

We aimed to validate the system control in canine HF models under the real-world disturbances.

The main protocol included five consecutive experiments in three tachycardia-induced HF animals, where we examined how accurately the system controlled the three hemodynamic variables to within *±*10% of their targets. The supplemental protocol included two experiments conducted in a single animal of tachycardia-induced HF complicated by coronary embolization, where we examined control limitations and their dependence on control targets.

In each experiment, five discrete drug interventions were applied to the animal to regulate the three metrics toward their targets.

## II. Results

A representative experimental time trace is shown in Fig. 2, which illustrates five discrete drug interventions that span approximately one hour, with continuous measurements of *MAP*, $P_{RA}$, and *PAP*, alongside discrete measurements of cardiac index (*CI*) and $P_{LA}$ via PAC. Let *CI* denote $y_{2}^{*}$, which is $y_{2}$:*CO* normalized to body surface area, providing a clinically relevant index for scaling *CO* across individuals. The hemodynamic variables *MAP*, *CI*, and $P_{LA}$ gradually converge toward their target values, $MAP_{d}=85$ mmHg, $CI_{d}=4.5$ L/min/m$^{2}$, and $P_{LA\_{d}}=10$ mmHg. These fixed-point targets were selected to facilitate quantitative evaluation of control performance while remaining within clinically acceptable hemodynamic ranges.

**Figure 2.**
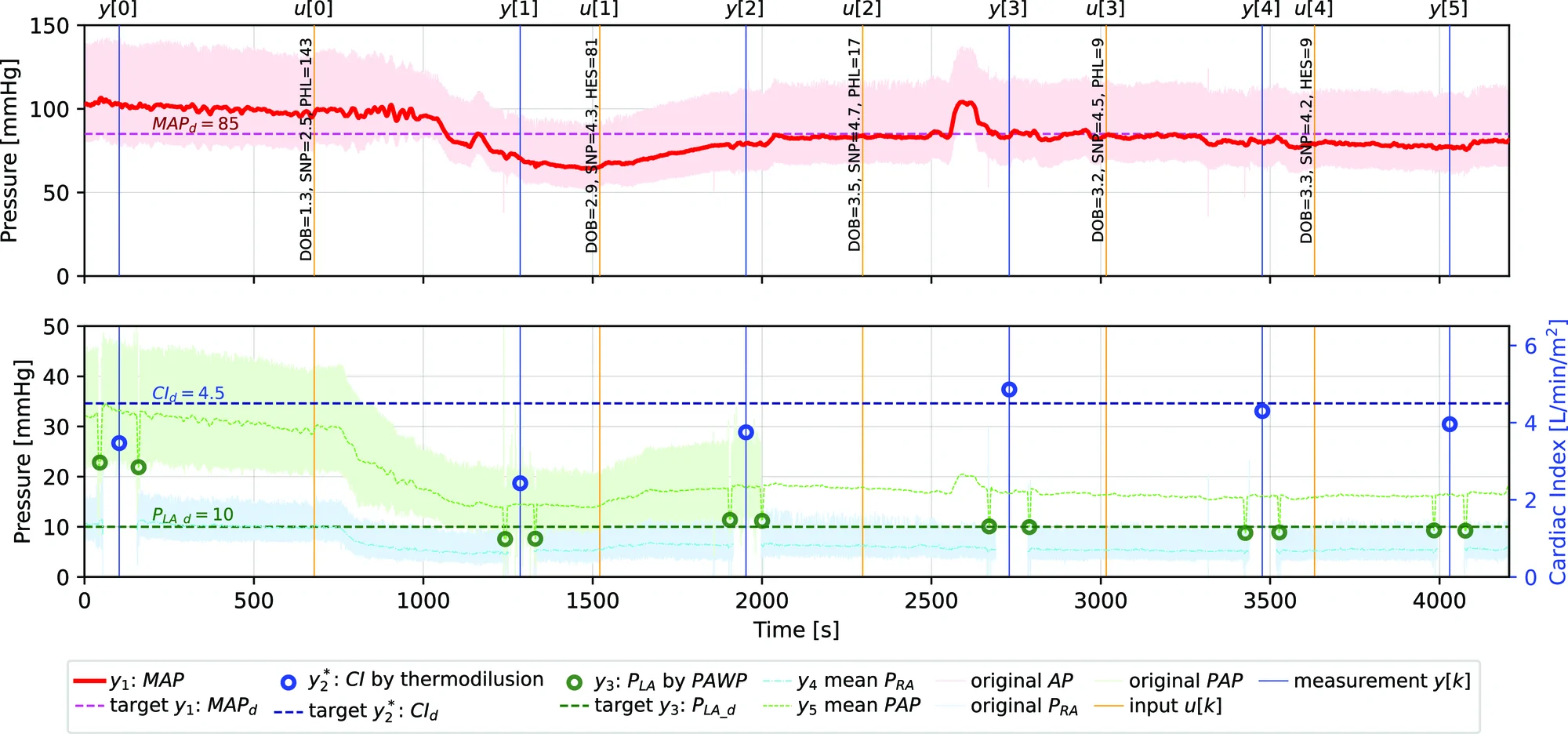
Representative Experimental Data. $y_{1}$:*MAP*, continuously monitored by arterial line. $y_{2}^{*}$:*CI*, discretely measured via thermodilution using a PAC. $y_{3}$:$P_{LA}$, discretely estimated from *PAWP* measurements obtained via PAC. $y_{4}$:$P_{RA}$, continuously monitored via PAC. $y_{5}$:*PAP*, continuously monitored via PAC. Note: Manual measurements ($y_{2}$, $y_{3}$) were performed after a 10-min observation. Due to manual procedures, the time intervals between discrete steps were not strictly uniform.

### A. Main Protocol

The hemodynamic variables *\bm y* measured in the main protocol are summarized in Fig. 3. Based on cohort-averaged *CI* and $P_{LA}$ in Fig. 3 (b), the baseline hemodynamics corresponded to Subset II in the *Forrester Classification* [12].

**Figure 3.**
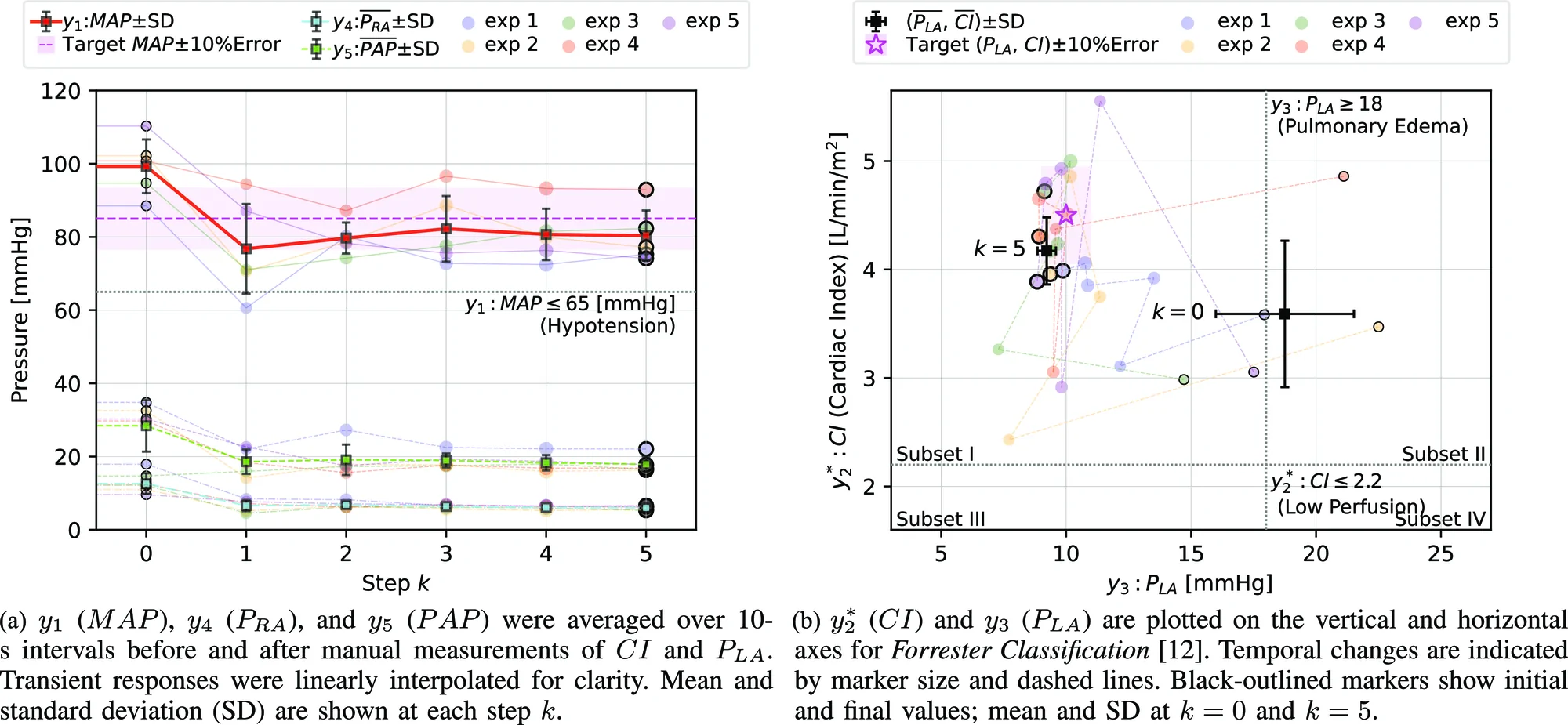
Results of *hemodynamic variables* $\bm {y}[k]$ in main protocol

The control performance metrics are indicated in Table 1. For evaluating the control performance, absolute percentage errors (APE [%] $:=\frac{|y[k]-y_{d}|}{y_{d}}\times 100$) were calculated for *MAP*, *CI*, and $P_{LA}$, allowing each convergence performance to be evaluated. Given the limited sample size, results are reported descriptively (mean *±* standard deviation) without formal inferential statistical testing.

**TABLE 1:** Performance Results for the Main Protocol. APE: Absolute Percentage Error. Before: Baseline At *k=0*. After: Average of the Measurements Obtained At *k=3*–5. $\min _{t}$ or $\max _{t}$: Extrema of Continuous Measurements. $\min _{k\geq 1}$ or $\max _{k\geq 1}$: Extrema of Post-Intervention Measurements.

	APE for *MAP*		APE for *CI*		APE for $P_{LA}$		*MAP(t)*		*CI[k]*	$P_{LA}[k]$	*HR(t)*	
EID	before	after	before	after	before	after	min$_{t}$	max$_{t}$	min$_{k\geq 1}$	max$_{k\geq 1}$	min$_{t}$	max$_{t}$
exp1	4.1%	13.5%	20.4%	11.9%	79.3%	5.0%	59.7	93.1	3.1	13.5	96	122
exp2	20.2%	3.6%	22.9%	2.8%	125.0%	4.8%	64.3	107.0	2.4	11.4	117	149
exp3	11.4%	5.3%	33.7%	3.4%	47.2%	3.4%	66.3	88.9	3.3	10.2	121	149
exp4	18.5%	10.9%	8.0%	0.2%	111.1%	7.1%	73.9	106.8	3.1	10.1	70	155
exp5	29.8%	11.4%	32.1%	0.8%	75.1%	7.2%	69.9	110.3	2.9	11.4	96	127
Mean*±*SD	16.8*±*9.7%	9.0*±*4.2%	23.4*±*10.4%	3.8*±*4.7%	87.5*±*30.9%	5.5*±*1.6%	66.8*±*5.4	101.2*±*9.6	3.0*±*0.3	11.3*±*1.4	105.2*±*22.3	135.2*±*24.4

From the baseline to the average of the third to fifth intervention results, the cohort-averaged APEs of *MAP*, *CI*, and $P_{LA}$ decreased from 16.8% to 9.0%, 23.4% to 3.8%, and 87.5% to 5.5%, respectively, indicating that each cohort-averaged variable was regulated to within *±* 10% of its target. Achieving simultaneous regulation of *MAP*, *CI*, and $P_{LA}$ across all five experiments would require relaxing the convergence criterion to *±*13.5%.

During the transient response following treatment initiation, hypotension (*MAP < 65* mmHg [13]) was observed in 2 of the 5 experiments. In contrast, low perfusion (*CI < 2.2* L/min/m$^{2}$ [12]) and pulmonary congestion ($P_{LA} > 18$ mmHg [12]) were not observed after treatment initiation (supplementary materials S-III).

The changes in input $\bm {u}$ and state $\bm {x}$ in the main protocol are shown in Fig. 4. Despite uniform HF induction across subjects, different drug combinations and dosages were used.

**Figure 4.**
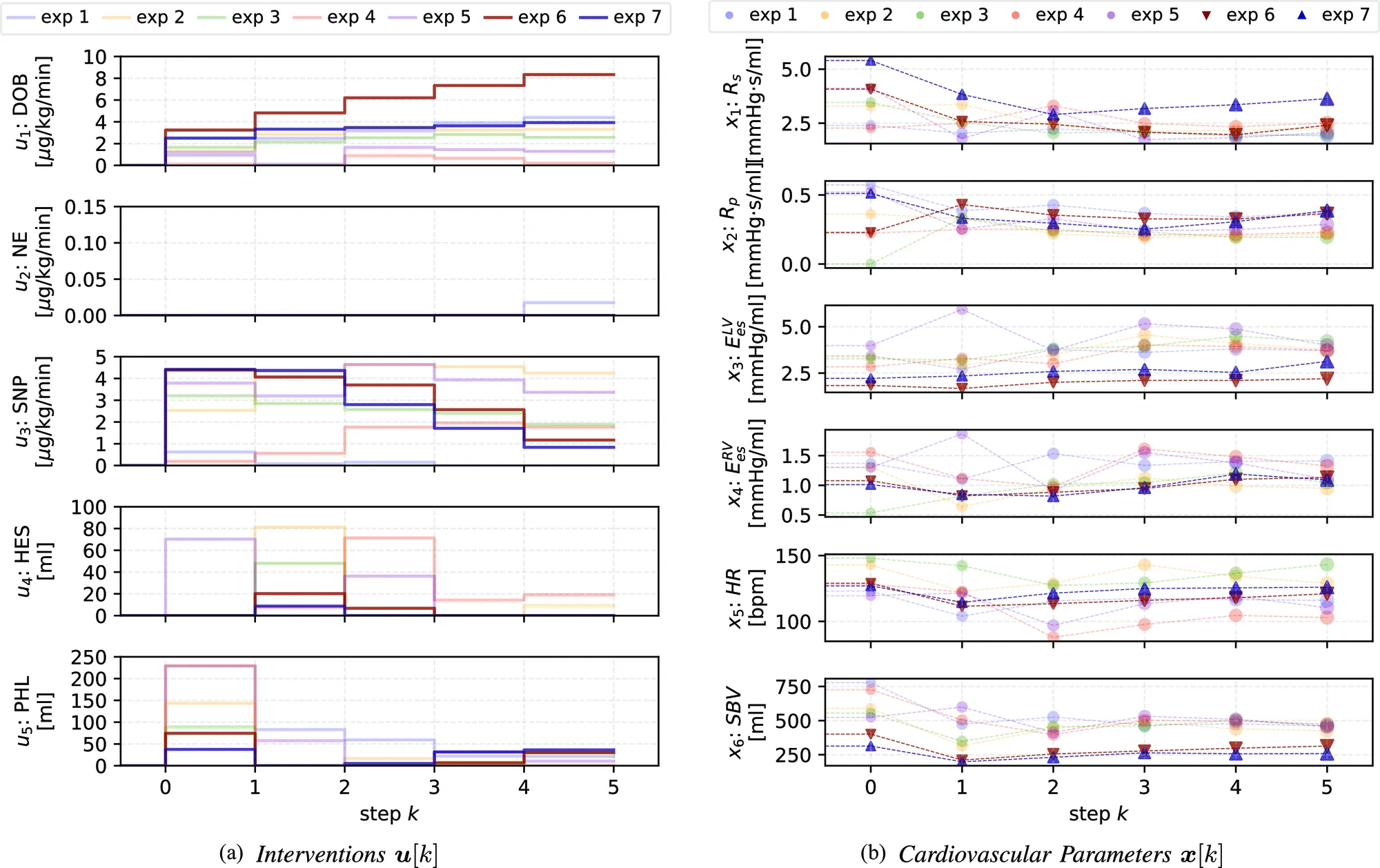
Results of *Interventions* $\bm {u}[k]$ and $\bm {x}[k]$ in both main and supplemental protocol

### B. Supplemental Protocol

The hemodynamic variables *\bm y* in the supplemental protocol are shown in Fig. 5. In both experiments, the hemodynamics at baseline was classified as Subset IV in the *Forrester Classification*, indicating more severe HF condition than the cases in the main protocol. The corresponding changes in input $\bm {u}$ and state $\bm {x}$ are shown in Fig. 4.

**Figure 5.**
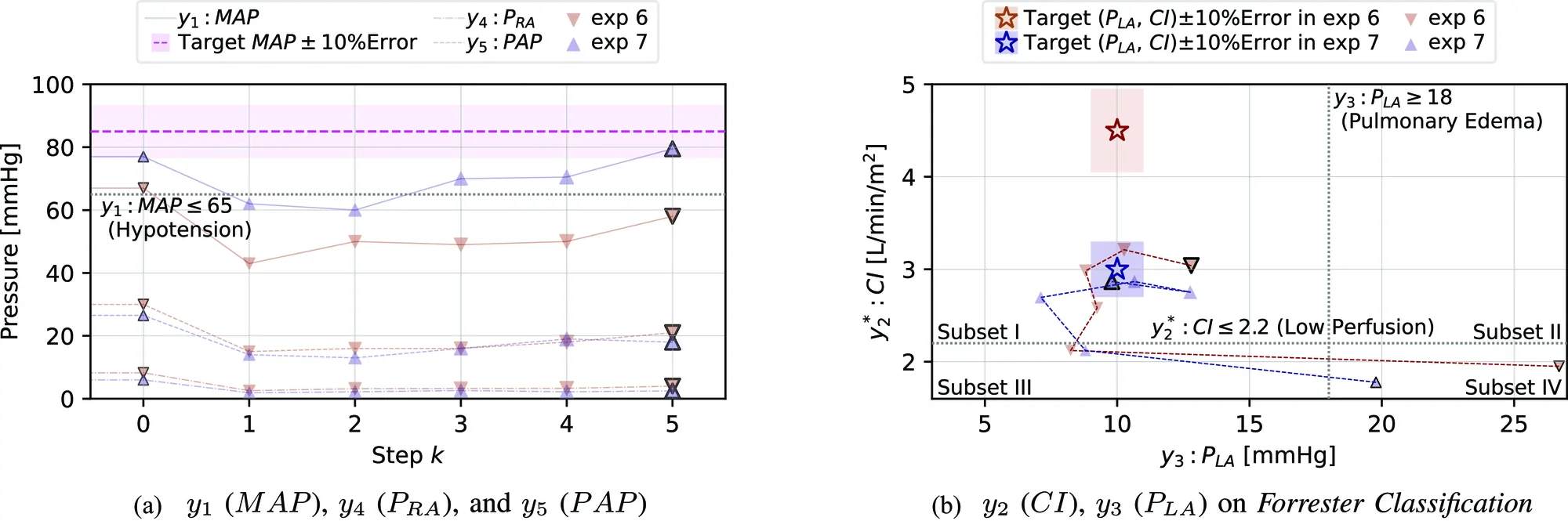
Results of *hemodynamic variables* $\bm {y}$ in supplemental protocol:severe heart failure case

In exp 6, when target *CI* was set to 4.5L/min/m$^{2}$, the system failed to achieve convergence within the 10% error range even though the system continuously increased inotropes $u_{1}$:*DOB*. In contrast, in exp 7, when target *CI* was reset to 3.0L/min/m$^{2}$, all three hemodynamic variables (*MAP*, *CI*, and $P_{LA}$) converged within 10% of their respective target values. Clearly in exp 6, the left ventricular contractility, $x_{3}$:$E_{es}^{LV}$, remained suppressed and did not improve in response to the inotropes. These results suggest that further optimization, including appropriate selection of the target value, is required especially under complex pathophysiological conditions.

## III. Discussion

This study presents the first in-vivo validation of the performance of the discrete 2DoF hemodynamic control system using PAC. With this clinically established but intermittent measurement modality of *CI* and $P_{LA}$, the system achieved simultaneous regulation of *MAP*, *CI*, and $P_{LA}$ in a clinically likely setting, demonstrating a significant advance toward its clinical implementation.

### A. Clinical Interpretation and Implications

#### 1) Measurement Feasibility

By utilizing PAC, routinely available in ICUs and CCUs, the system worked effectively under realistic discrete hemodynamic measurements and cardiovascular parameter estimation methods [14], [15] (Supplementary Materials S-IV).

#### 2) Robust Control Performance

Despite the control algorithm relying on a simplified analytical model with a provisional drug library, the system regulated hemodynamics within *±*10% of targets across multiple interventions, demonstrating robustness in in-vivo conditions against model discrepancies, as well as measurement and estimation errors. These results validate feedback compensation around approximate drug-effect directions rather than the specific entries of the drug library matrix.

#### 3) Interpretability and Transparency

The control strategy of the system is based on established cardiovascular causality, allowing prediction of intermediate physiological states and outputs (supplementary materials V). This enables identification of causes of control failure (e.g. less sensitivity of $u_{1} \rightarrow x_{3}$ in exp 6), providing transparency, debuggability, and clinical accountability.

#### 4) Potential for Treatment Recommendation Tool

By updating interventions only after hemodynamic assessment at each discrete step, the proposed framework is compatible with the clinical workflow in which treatment decisions are revised based on newly obtained hemodynamic information. Thus, the proposed system is intended to initially function as a clinical decision-support tool, allowing treatment recommendations to be incorporated into routine clinical assessments [16].

### B. Main Protocol

#### 1) *MAP* Control

During control, *MAP* often remained below $MAP_{d}$, likely due to physiological effects not captured by the model. In most trials (except exp 5), phlebotomy-induced *SBV* reduction appeared to trigger the *Bezold–Jarisch reflex* [17], reducing *HR*, *CI*, and consequently *MAP*. Although this reflex was not modeled, its effect was compensated for by MIMO feedback control.

For example, to compensate for the reduction in *MAP*, increasing $R_{s}$ or *SBV* could adversely affect *CI* or $P_{LA}$, respectively, whereas increasing $E_{es}^{LV}$ improves both *MAP* and *CI* without worsening $P_{LA}$.

Representative examples are exp 1 (*k=1 → 2*) and exp 4 (*k=2 → 3*) shown in Fig. 4. In exp 1, the controller selected *DOB* and *PHL*, whereas in exp 4 it selected *DOB*, *SNP*, and *HES*. These distinct interventions illustrate how the MIMO feedback controller selected different therapeutic strategies according to the hemodynamic condition.

Remaining challenges include the system's inability to respond during transients, allowing short hypotensive episodes. To address this, we proposed a hybrid hemodynamic control strategy that continuously prioritizes *MAP* while adjusting *CO* and $P_{LA}$ discretely [18].

#### 2) *CI* Control

*CI* was affected by *HR* reduction. In exp 4, *HR* dropped from 122.4 to 87.95 bpm between *k=1* and *k=2*, reducing *CI* from 4.34 to 3.05 L/min/m$^{2}$. The system responded with large adjustments in *DOB*, *SNP*, and *HES* at the next step, restoring *CI* near the target at *k=3* despite only partial recovery of *HR*.

Measurement noise and rounding errors caused variability. In thermodilution, the mean standard deviation of the three lowest-variance samples was 0.125 L/min/m$^{2}$ ($\approx \pm 2.8$% of $CI_{d}=4.5$). The device's one-decimal display of *CO* introduced rounding errors up to *±*0.11 L/min/m$^{2}$ ($\approx \pm 2.4$% of $CI_{d}$). Despite these measurement errors, low perfusion (*CI ≤ 2.2* L/min/m$^{2}$) was avoided in all cases in the main protocol.

#### 3) $P_{LA}$ Control

According to the circulatory equilibrium framework [11], $P_{LA}$ can be lowered by reducing *SBV* and enhancing the cardiac function curve. In this study, interventions explicitly incorporating this principle demonstrated highly precise $P_{LA}$ control.

Averaging two *PAWP* measurements caused measurement variability. Across all experiments, the mean standard deviation was 0.32 mmHg ($\approx \pm 3.2$% of $P_{LA\_{d}}$ = 10 mmHg). Despite these measurement errors, pulmonary edema ($P_{LA} \geq 18$ mmHg) was avoided in all cases in the main protocol.

Phlebotomy was used instead of diuretics to reduce experimental variability and validate the concept. In clinical practice, diuretic effects depend on renal function and transrenal pressure gradient. Future studies should incorporate diuretics and their variability.

### C. Supplemental Protocol

#### 1) Strategies in Limited Pharmacological Therapy

In exp 6, cardiac contractility did not improve despite increased dose of inotrope, indicating drug resistance. The new target was manually selected based on the observed pharmacological limitation in exp 6. In future systems, poor drug responsiveness and reachable hemodynamic changes should ideally be identified proactively to support adaptive target setting. In exp 7, control was achieved by lowering the *CI* target; however, higher $P_{LA}$ target settings may also contribute to higher *CI* according to the *Frank-Starling* principle.

Overall, the results highlight the importance of adaptive target setting. Future supervisory logic may assess target feasibility, identify unreachable objectives, and dynamically redefine target regions according to the patient's physiological condition and therapeutic priorities.

#### 2) Strategies to Avoid Coronary Hypoperfusion

In the supplemental protocol, the initial *MAP* was low. The current system treats *MAP*, *CI*, and $P_{LA}$ with equal priority, using *SNP* to reduce afterload for *CI* improvement and using phlebotomy to decrease *SBV* for $P_{LA}$ improvement. While both interventions are reasonable for *CI* and $P_{LA}$, these decrease *MAP*, risking coronary hypoperfusion. Experimentally, coronary arterial pressure below 67.0 mmHg reduces $E_{es}^{LV}$ [19], further worsening *CI* and complicating hemodynamic control. For clinical application, the system will need to be fine-tuned to prioritize *MAP*, and target trajectories for $\bm {y}_{1:3}$ should be designed to avoid abrupt reductions.

### D. Other Limitations

#### 1) Uniform Drug Library

Pharmacological responses are subject to inter- and intra-individual variability as well as nonlinear dose–response characteristics. Real-time identification of the drug library is desirable for achieving more individualized and accurate hemodynamic control [20]. For clinical translation, the canine-derived drug library will require allometric scaling and calibration using human physiological and pharmacological data.

#### 2) Phlebotomy Instead of Diuretics

Decreasing *SBV* was precisely achieved via phlebotomy as a proof-of-concept study, although this approach is not used in standard HF treatment. In clinical practice, variability in diuretic responsiveness may compromise *SBV* and consequently $P_{LA}$ regulation. Future in-vivo studies should therefore incorporate diuretic therapy.

#### 3) Convergence Speed

One disadvantage of the discrete control compared with continuous feedback control is its slower convergence. Therefore, assessing the number of intervention updates required for effective hemodynamic control is critical. However, because phlebotomy rather than diuretics was used in the present experiments, drawing definitive conclusions from this study is not warranted.

#### 4) Convergence Performance

The clinical goals for optimizing *MAP*, *CI*, and $P_{LA}$ are range-based, and the present results meet these criteria. Implementing more complex tasks in in-vivo conditions, including minimization of myocardial oxygen consumption ($MVO_{2}$) [10], [20], may require the system to achieve more precise hemodynamic regulation, making fine-tuning indispensable.

Future extensions may explicitly incorporate physiological reflexes, such as the *Bezold–Jarisch* reflex, into the predictive model. However, whether increased physiological fidelity improves control performance without compromising robustness remains an open question.

#### 5) Sample Size and Diversity

As a proof-of-concept study, the limited sample size precludes assessment of inter-subject variability and generalizability. Larger studies are needed to evaluate these aspects.

## IV. Conclusion

This study presents the first in vivo validation of a discrete two-degree-of-freedom hemodynamic control system regulating *MAP*, *CO*, and $P_{LA}$ by titrating the cardiovascular drugs under the pulmonary artery catheter monitoring. In tachycardia-induced HF canine models, cohort-averaged hemodynamic variables were regulated to each within *±*10% of their respective targets after five interventions despite real-world uncertainties, including pharmacological variability, model discrepancies, and PAC-related measurement noise. Control performance varied in a more complex HF model with coronary embolization, highlighting the need for tuning such as target value selection. These findings provide initial in-vivo evidence supporting the feasibility of closed-loop hemodynamic control in anesthetized tachycardia-pacing canine HF under PAC monitoring and phlebotomy-mediated volume removal. Future work should assess generalizability and incorporate real-world variability, including diuretic response.

## Materials and Methods

### A. In-Vivo Experiments

#### 1) Main & Supplemental Protocols

In the main protocol, the objective was to evaluate control performance in simultaneously regulating *MAP*, *CI*, and $P_{LA}$, with target values of 85mmHg, 4.5L/min/m$^{2}$, and 10 mmHg, respectively, over five intervention updates. Five consecutive experiments were conducted in three canine models of tachycardia-induced HF.

In the supplemental protocol, the objective was to demonstrate the limitations of the control system and pharmacological intervention, and their dependence on the target values. Two consecutive experiments were conducted in a canine model of severe HF induced by tachycardia and coronary embolization.

A total of seven experiments were conducted in four canines as described in Table 2.

**TABLE 2:** Experiment Setups; EID = Experimental ID, AID = Animal ID, $CI_{d}$ = Target *CI* Value

EID	mark	protocol	AID	init. Subset	$CI_{d}$
exp 1	*∘*	main	a	Subset I	4.5
exp 2	*∘*	main	b	Subset II	4.5
exp 3	*∘*	main	b	Subset I	4.5
exp 4	*∘*	main	c	Subset II	4.5
exp 5	*∘*	main	c	Subset I	4.5
exp 6	*▿*	supplement	d	**Subset IV**	4.5
exp 7	*△*	supplement	d	**Subset IV**	**3.0**

#### 2) Animal Preparation

Animal housing and experimental procedures followed the ARRIVE guidelines [21] and the Guiding Principles for the Care and Use of Animals in the Field of Physiological Sciences [22] approved by the Physiological Society of Japan. All protocols were reviewed and approved by the Animal Subjects Committee at the National Cerebral and Cardiovascular Center (Permission numbers:24020).

The specifications of the four beagle dogs (Kitayama Labes Ltd, Gifu, Japan) [female/male = 1/3, body weight *10 ± 1* kg, age 1 years]. For all, HF was induced by pacing the heart at 240 in bpm starting 28 days before the experiment. On the experimental day, animals were anesthetized, intubated, and mechanically ventilated with continuous isoflurane inhalation. Isoflurane concentration was manually adjusted to ensure *MAP ≤ 110* only once before the experiment to prevent unintended emergence from anesthesia. Animals were placed supine. A surface ECG was recorded to monitor *HR*. Arterial pressure was measured via a 22 G catheter inserted into the right radial artery and connected to a pressure transducer (DX-200, Nihon Kohden, Tokyo, Japan). An 8-Fr catheter was placed in the right femoral vein for drug/fluid administration and another in the left femoral vein for phlebotomy. A 8-Fr catheter was inserted into the left internal jugular vein, through which a 5-Fr PAC (Edwards LifeSciences) was introduced. Zero pressure was referenced to the junction of the vena cava and right atrium [23]. One out of the four animals underwent coronary embolization, causing partial occlusion of a coronary artery and inducing acute myocardial infarction for the supplemental protocol. Trans-thoracic ultrasound echo-cardiography revealed that left ventricular ejection fraction decreased from *59.0 ± 4.1* % before the pacing to *34.0 ± 3.5* % at the experiment (supplementary materials S-VI), indicating that the pacing with/without the coronary embolization successfully induced HF. The complete setup is shown in Fig. 6.

**Figure 6.**
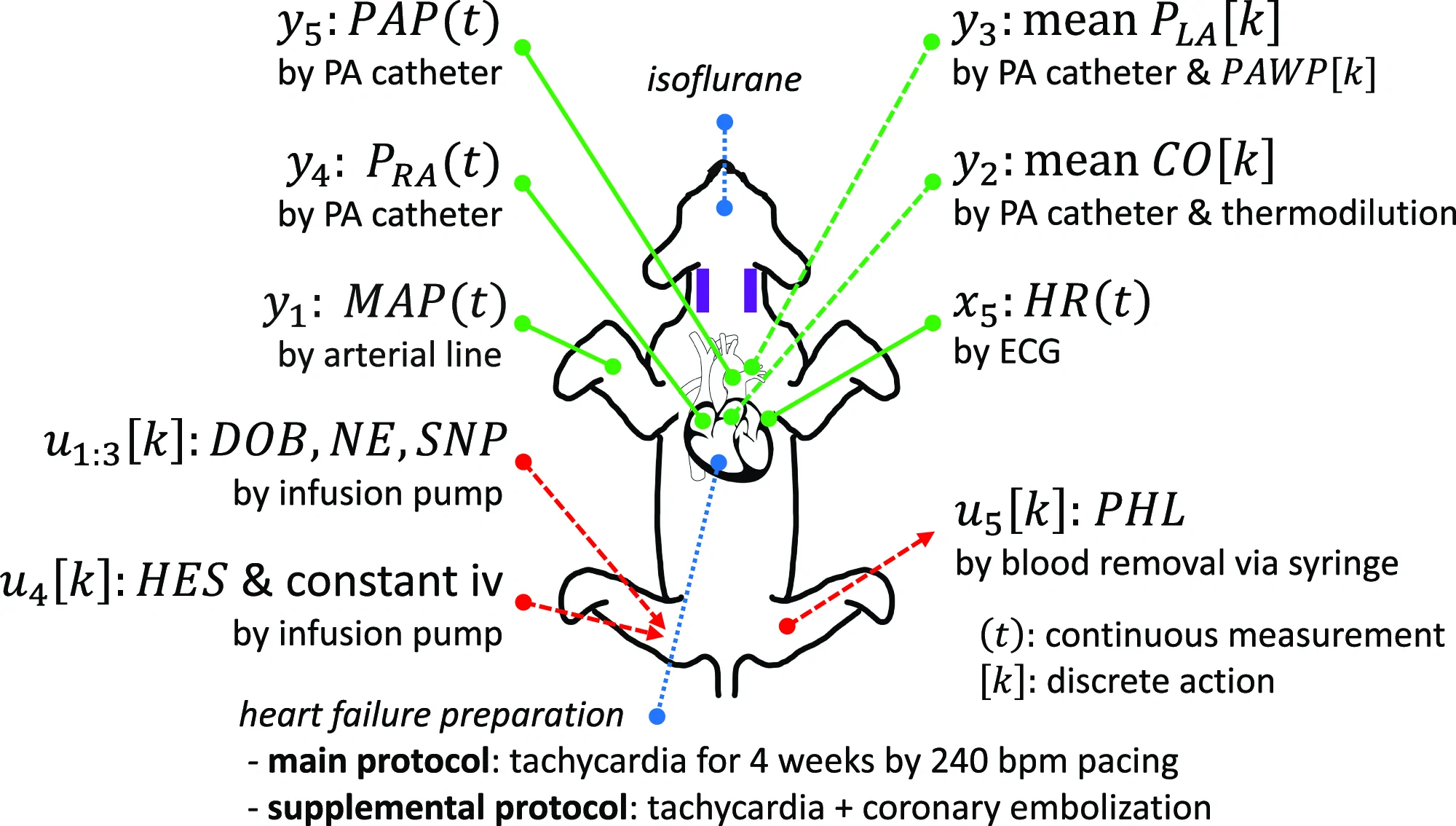
Experiment Setup for Canine Model.

For repeated experiments in the same animal, drug infusions were discontinued and a washout/stabilization period of at least 1 h was allowed. Blood removed by phlebotomy was reinfused before the subsequent experiment. Because hemodynamic conditions were not expected to return to identical baseline values, repeated experiments were treated as distinct control scenarios while following the principles of the 3Rs (Replacement, Reduction, and Refinement) in animal research.

#### 3) Measurement and Estimation

This setup allowed continuous measurement of $y_{1}$:*MAP(t)*, $y_{4}$:$P_{RA}(t)$, $y_{5}$:*PAP(t)* and $x_{5}$:*HR(t)*. Using PAC, $y_{2}$:*CO[k]* by thermodilution with 5ml cold saline bolus injection and $y_{3}$:$P_{LA}[k]$ by *PAWP* are obtained discretely.

The states $\bm {x}$ were estimated based on these measurements $\bm {y}$. The systemic and pulmonary resistances, $x_{1}$:$R_{s}[k]$ and $x_{2}$:$R_{p}[k],$ were discretely computed by basic vascular resistance model. The left and right ventricular elastances, $x_{3}$:$E_{es}^{LV}[k]$ and $x_{4}$:$E_{es}^{RV}[k]$, were estimated using inverse end-systolic pressure volume relationship estimation method [14]. The stressed blood volume, $x_{6}$:*SBV*, was estimated using the venous return surface [15]. (supplementary materials S-IV)

#### 4) Intervention

The inputs $\bm {u}[k]$ computed by the 2DoF hemodynamic control system were administered as follows. The inputs $u_{1}$:*DOB*, $u_{2}$:*NE*, and $u_{3}$:*SNP* were delivered via an electronically controlled syringe pump (Terufusion$^{\mathrm{TM}}$, TERUMO, Japan) at constant rates in *μ*g/min/kg through a right femoral vein catheter. Fluid infusion for volume elevation $u_{4}$:*HES* and phlebotomy for volume reduction $u_{5}$:*PHL* were performed manually via the femoral vein at approximately 30 mL/min. Phlebotomy was chosen instead of diuretics for its rapid onset, precision, and independence from renal function, enabling rigorous evaluation of the control system under idealized conditions.

The 2DoF control scheme was applied with the parameter settings in two phases. (supplementary materials S-I & S-II) At the initial step (*k=0* to *k=1*), substantial hemodynamic changes were induced while avoiding overshoot and undershoot using feedforward control, with 70% of the current-to-target gap set as an intermediate goal. In the subsequent phase (*k ≥ 1*), hemodynamics were gradually driven toward the target values using feedback control, as changes were smaller. Drug-specific upper dose limits are imposed as optimization constraints (S-15), and infeasibility indicates that the target is unreachable within the predefined dose limits.

#### 5) Control Experiment Procedure

Discrete recordings were performed in the following sequence:

1) 10-s averages of *MAP*, $P_{RA}$, *PAP*, and *HR*;
2) 10-s average of *PAWP* to estimate $P_{LA}$;
3) Four thermodilution measurements of *CO*, with the three most consistent values averaged;
4) 10-s average of *PAWP* to estimate $P_{LA}$;
5) 10-s averages of *MAP*, $P_{RA}$, *PAP*, and *HR*;
6) Averaging these values for $\bm {y}$ at the step;
7) Estimation of the cardiovascular state $\bm {x}$ at the step;
8) Administration of the optimal input $\bm {u}$ at the step;
9) 10-min waiting period for physiological stabilization;
10) Steps 1–9 were repeated until step *k=5*.

Post-thermodilution measurements were performed to account for changes in *SBV* from four 5 mL cold-saline injections and to calculate the average hemodynamics before and after the 20 mL infusion. *MAP*, $P_{RA}$, *PAP*, *PAWP*, and *HR* were recorded using LabChart (ADInstruments Ltd., Dunedin, New Zealand), while *CO* measured by thermodilution was manually entered into the same tool.

## Appendix

Supplementary materials provided online include:

I. 2DoF Hemodynamic Control System
II. Parameter values of the model and the controller
III. Experimental data of *CI* and $P_{LA}$
IV. Discrete *CV Parameters* $\bm {x}$ estimation methods
V. Visualization tool for hemodynamic control
VI. Left Ventricular Ejection Fraction before and after HF induction

along with a nomenclature.

Supplementary Materials
