# Supplementary material for: A Discrete Hemodynamic Control System: Proof-of-Concept In-Vivo Study in Canine Heart Failure Using a Pulmonary Artery Catheter
Source: IEEE Open J Eng Med Biol. 2026 Aug 12;7:332–40. doi: 10.1109/OJEMB.2026.3723319 (PMC13667294; doi:10.1109/OJEMB.2026.3723319)
Supplement: Supplementary file 1 [file supp1-3723319.pdf]

Supplementary Materials - Nomenclature

A Discrete Hemodynamic Control System: Proof-of-Concept In-Vivo Study in Canine Heart Failure Using a Pulmonary Artery Catheter

Yasuyuki Kataoka, *Member, IEEE*, Kazunori Uemura,Takuya Nishikawa, *Member, IEEE*, Yukiko Fukuda, *Member, IEEE*, Jon Peterson, *Senior Member, IEEE*, Kei Sato, Hiroki Matsushita, Hidetaka Morita, Yuta Nakamura, Masahiro Otake, Kenta Ohba, Nana Hiraki, Keita Saku, Mitsuji Sampei, *Life Member, IEEE*, Joe Alexander Jr, and Kenji Sunagawa, *Life Fellow, IEEE*

**Term**	**Abbreviation**	**Meaning**
Absolute Percentage Error	APE	Absolute percentage difference between measured and targeted value
Cardiac Index	CI	Cardiac output normalized by body surface area
Cardiac Output	CO	Volume of blood pumped by the heart per minute
Circulatory Equilibrium	–	Balance point of the cardiovascular system where cardiac output equals venous return
Coronary Care Unit	CCU	Specialized unit for patients with severe cardiovascular disease
Dobutamine	*DOB*	A positive inotropic agent
Forrester Classification	–	Hemodynamic classification of patients with acute heart failure
Heart Rate	HR	Number of heart beats per minute
Hydroxyethyl Starch	*HES*	A colloid fluid
Intensive Care Unit	ICU	Specialized unit for critically ill patients
Left Atrial Pressure	P_{LA}	Pressure within the left atrium
Left Ventricular End-Systolic Elastance	E_{es}^{LV}	Index of left ventricular contractility
Mean Arterial Pressure	MAP	Mean pressure in the systemic arterial circulation
Norepinephrine	*NE*	A vasopressor
Phlebotomy	*PHL*	Removal of blood from the circulation
Pulmonary Artery Catheter	PAC	Catheter used to measure hemodynamic variables such as CO, P_{RA}, PAP and $PAWP$
Pulmonary Artery Pressure	PAP	Pressure within the pulmonary artery
Pulmonary Artery Wedge Pressure	$PAWP$	Indirect estimate of left atrial pressure measured using a PAC
Pulmonary Vascular Resistance	R_p	Resistance of the pulmonary circulation
Right Atrial Pressure	P_{RA}	Pressure within the right atrium
Right Ventricular End-Systolic Elastance	E_{es}^{RV}	Index of right ventricular contractility
Sodium Nitroprusside	*SNP*	A vasodilator
Stressed Blood Volume	SBV	Blood volume contributing to venous return
Systemic Vascular Resistance	R_s	Resistance of the systemic circulation
Two-Degree-of-Freedom Control	2DoF	Control method consisting of feedforward and feedback components
