# Supplementary material for: A Discrete Hemodynamic Control System: Proof-of-Concept In-Vivo Study in Canine Heart Failure Using a Pulmonary Artery Catheter
Source: IEEE Open J Eng Med Biol. 2026 Aug 12;7:332–40. doi: 10.1109/OJEMB.2026.3723319 (PMC13667294; doi:10.1109/OJEMB.2026.3723319)
Supplement: Supplementary file 2 [file supp2-3723319.pdf]

Supplementary Materials

A Discrete Hemodynamic Control System: Proof-of-Concept In-Vivo Study in Canine Heart Failure Using a Pulmonary Artery Catheter

Yasuyuki Kataoka, *Member, IEEE*, Kazunori Uemura,Takuya Nishikawa, *Member, IEEE*, Yukiko Fukuda, *Member, IEEE*, Jon Peterson, *Senior Member, IEEE*, Kei Sato, Hiroki Matsushita, Hidetaka Morita, Yuta Nakamura, Masahiro Otake, Kenta Ohba, Nana Hiraki, Keita Saku, Mitsuji Sampei, *Life Member, IEEE*, Joe Alexander Jr, and Kenji Sunagawa, *Life Fellow, IEEE*

S-I. 2DoF Hemodynamic Control System

A versatile discrete hemodynamic control framework has been proposed [10]. In the present study, the controller is tailored to regulate three-dimensional targets, specifically MAP, CO, and P_{LA} using slightly different intervention. This part articulates the setup for the control system used in this study.

A. System Representation

Let *intervention* $\boldsymbol{u}$ be the input $\boldsymbol{u} := [u_1, \cdots, u_5]^T \in \mathbb{R}^5$ as shown in Fig. 1. The therapeutic drugs were selected to represent key classes for heart failure: Dobutamine (*DOB*) in μg/kg/min as a positive inotrope, Norepinephrine (*NE*) in μg/kg/min as a vasopressor, Sodium Nitroprusside (*SNP*) in μg/kg/min as a vasodilator, and Hydroxyethyl Starch (*HES*) in ml as a fluid. In liue of diuretic, phlebotomy (*PHL*) in ml is used in this study.

Let *cardiovascular parameters (CV parameters)* $\boldsymbol{x}$ be the state representing the primary factors that affect hemodynamics: $\boldsymbol{x} := [R_s, R_p, E_{es}^{LV}, E_{es}^{RV}, HR, SBV]^T \in \mathbb{R}^6$ where R_s and R_p are systemic and pulmonary vascular resistance in mmHg·s/ml, E_{es}^{LV} and E_{es}^{RV} are left and right ventricular cardiac contractility represented by end-systolic elastance in mmHg/ml, HR is heart rate in beat/min, and SBV is stressed blood volume in ml.

Let *hemodynamics* $\boldsymbol{y}$ be the output defined as $\boldsymbol{y} := [MAP, CO, P_{LA}, P_{RA}, PAP]^T \in \mathbb{R}^5$ where MAP is mean systemic arterial pressure in mmHg, CO is cardiac output in ml/min, P_{LA} and P_{RA} are mean left and right atrial pressure in mmHg, and PAP is mean pulmonary arterial pressure in mmHg. For clinical interpretation of treatment experiments, cardiac output (CO) was converted to cardiac index (CI) using $CI = \frac{CO}{1000BSA}$ in L/min/m^2, where BSA is the body surface area in m^2 calculated from body weight.

As for cardiovascular causality shown in Fig. 1, *intervention* $\boldsymbol{u}$ directly affects *CV parameters* $\boldsymbol{x}$ from the patient's initial state $\boldsymbol{x}[0]$ based on their pharmacologic properties. Then, the changes in $\boldsymbol{x}$ will in turn affect *hemodynamics* $\boldsymbol{y}$. The resulting $\boldsymbol{y}$ converges at the point called circulatory equilibrium.

B. Drug Infusion Model

The drug infusion model represents the pharmacological effects of multi-dependencies among multiple *intervention* $\boldsymbol{u}$ against *CV parameters* $\boldsymbol{x}$ when drug effects have converged. The model assumptions are a) each drug functions to add its effects linearly to influence the patient's initial *CV parameters* $\boldsymbol{x}[0]$ and b) cross-terms of the multiple inputs, e.g., drug interaction such as u_1u_2, are not considered.

The discrete drug infusion model can be represented by

$$\boldsymbol{x}[k+1] = \boldsymbol{B}\boldsymbol{u}[k] + \boldsymbol{x}[0] \tag{S-1}$$

where $\boldsymbol{x}[k+1]$ shows the *CV parameters* following drug infusion $\boldsymbol{u}[k]$, once updated and converged. Various factors affect the duration to converge steady state, such as the dead time, time constant, and half-life of each drug. Thus, the time duration for each step k varies depending on the input selection, which may range from minutes to hours. In this experiment, a 10 minute was set. The input matrix $\boldsymbol{B} \in \mathbb{R}^{6\times5}$ is the *drug library* that represents the multi-dependency effect from each input to *CV parameters* $\boldsymbol{x}$. Each element $b_{ij} = (\boldsymbol{B})_{ij}$ is equivalent to the gain $(u_j \to x_i)$.

To determine the value of $\boldsymbol{B}$, preliminary experiments were conducted using healthy canine models. The identified values are in S-II-A.

C. Circulatory Equilibrium Framework

When dynamic response converges, the averaged hemodynamics stay at circulatory equilibrium, which is the state of balance in the circulatory system of the body, involving the heart, blood vessels, and blood.

Generalized circulatory equilibrium framework (*GCE*) [11] indicated that the circulatory equilibrium could be identified by the intersection of two of *Frank-Starling curved surface* of the right and left heart systems and the *venous return surface* (*VRS*). Adding the one-resistance model of systemic and pulmonary circulation for MAP and PAP, the circulatory equilibrium are represented as the implicit vector function $\boldsymbol{F}(\boldsymbol{x}, \boldsymbol{y}) = [f_1(\boldsymbol{x}, \boldsymbol{y}), \cdots, f_5(\boldsymbol{x}, \boldsymbol{y})]^T = \boldsymbol{0} \in \mathbb{R}^5$:

$$f_1 := y_1 - y_4 - \frac{y_2 x_1}{60} \tag{S-2}$$

$$f_2 := y_5 - y_3 - \frac{y_2 x_2}{60} \tag{S-3}$$

$$f_3 := -y_2 + \frac{1}{\beta_{ed}^{LV}} \frac{x_3 x_5}{x_3 + x_1 \frac{x_5}{60}} g_l(y_3) - \frac{x_5}{x_3 + x_1 \frac{x_5}{60}} y_4 \tag{S-4}$$

$$f_4 := -y_2 + \frac{1}{\beta_{ed}^{RV}} \frac{x_4 x_5}{x_4 + x_2 \frac{x_5}{60}} g_r(y_4) - \frac{x_5}{x_4 + x_2 \frac{x_5}{60}} y_3 \tag{S-5}$$

$$f_5 := -y_2 + \left(\frac{SBV}{W} - G_s\, BW\, y_4 - G_p\, BW\, y_3\right). \quad \text{(S-6)}$$

where

$$g_l(P_{LA}) = \log\left(\frac{P_{LA}}{\alpha_{ed}^{LV}} + 1\right) + \beta_{ed}^{LV}(V_d^{LV} - V_0^{LV}) \quad \text{(S-7)}$$

$$g_r(P_{RA}) = \log\left(\frac{P_{RA}}{\alpha_{ed}^{RV}} + 1\right) + \beta_{ed}^{RV}(V_d^{RV} - V_0^{RV}), \quad \text{(S-8)}$$

f_1 and f_2 are the models of systemic and pulmonary circulation, f_3 and f_4 are the left-heart and right-heart *Frank-Starling curved surface*, and f_5 is *VRS*. All the parameter values used in the circulatory equilibrium framework is indicated in S-II-B.

In general, linearizing a nonlinear system enhances the clarity of the model representation, thereby facilitating analytical insight. Moreover, such linear approximations are particularly effective when output variations are small, as is often the case under feedback control near $\boldsymbol{y}_d$. The change of *hemodynamics* $\Delta\boldsymbol{y}$ with respect to the change of *CV parameters* $\Delta\boldsymbol{x}$ can be linearly represented by the gradient $\boldsymbol{G} \in \mathbb{R}^{5\times6}$ [10]. The gradient $\boldsymbol{G}$ can be derived using *implicit function theorem* as

$$\boldsymbol{G}(\boldsymbol{x},\boldsymbol{y}) = -\left(\frac{\partial \boldsymbol{F}(\boldsymbol{x},\boldsymbol{y})}{\partial \boldsymbol{y}}\right)^{-1} \frac{\partial \boldsymbol{F}(\boldsymbol{x},\boldsymbol{y})}{\partial \boldsymbol{x}} \quad \text{(S-9)}$$

$$\Delta\boldsymbol{y} = \boldsymbol{G}(\boldsymbol{x}[k],\boldsymbol{y}[k])\Delta\boldsymbol{x}. \quad \text{(S-10)}$$

Note that the gradient $\boldsymbol{G}$ can be analytically derived and its values change depending on the current *CV parameters* $\boldsymbol{x}[k]$ and *hemodynamics* $\boldsymbol{y}[k]$.

D. Control Methods

The 2DoF hemodynamic controller is composed of feedforward and feedback control as shown in Fig. 1. Given $\Delta\boldsymbol{u} = \boldsymbol{u}[k] - \boldsymbol{u}[k-1]$ $(\boldsymbol{u}[-1] = 0)$, $\Delta\boldsymbol{x} = \boldsymbol{x}[k+1] - \boldsymbol{x}[k]$, and $\Delta\boldsymbol{y} = \boldsymbol{y}[k+1] - \boldsymbol{y}[k]$, the control input is designed by solving the constrained optimization problem.

In feedforward control at $k = 0$ when the hemodynamic change $\Delta\boldsymbol{y}$ is expected to be large, the nonlinear *GCE* model should represent the hemodynamic change accurately. Thus, the optimal control can be computed by

$$\min_{\Delta\boldsymbol{u}} \quad \Delta\boldsymbol{u}^T \boldsymbol{W}_u \Delta\boldsymbol{u} \quad \text{(S-11)}$$

$$\text{s.t.} \quad \Delta\boldsymbol{x} = \boldsymbol{B}\Delta\boldsymbol{u} \quad \text{(S-12)}$$

$$\boldsymbol{F}(\boldsymbol{x}[k] + \Delta\boldsymbol{x}, \boldsymbol{y}[k] + \Delta\boldsymbol{y}) = \boldsymbol{0} \quad \text{(S-13)}$$

$$\Delta\boldsymbol{y}_{1:3} = -\boldsymbol{E}[k] \quad \text{(S-14)}$$

$$-\boldsymbol{u}[k-1] \leq \Delta\boldsymbol{u} \leq \overline{\boldsymbol{u}} - \boldsymbol{u}[k-1] \quad \text{(S-15)}$$

$$u_i[k]u_j[k] = 0 \text{ where } (i,j) \in S \quad \text{(S-16)}$$

$$\underline{\boldsymbol{x}} - \boldsymbol{x}[k] \leq \Delta\boldsymbol{x} \leq \overline{\boldsymbol{x}} - \boldsymbol{x}[k] \quad \text{(S-17)}$$

where the error of the target hemodynamics $\boldsymbol{E}[k] \in \mathbb{R}^3$ can be represented by P control logic as

$$e_i[k] = y_i[k] - y_{di} (i = 1,2,3), \boldsymbol{e}[k] \in \mathbb{R}^3 \quad \text{(S-18)}$$

$$\boldsymbol{E}[k] = \boldsymbol{K}_p \boldsymbol{e}[k]. \quad \text{(S-19)}$$

The objective function is set to minimize the drug modification where $\boldsymbol{W}_u$ is for normalizing the scale of dosages. The constraint conditions are for hemodynamic regulation, where (S-12) is the drug infusion model ($\boldsymbol{u} \to \boldsymbol{x}$), and (S-13) is *GCE* constraints ($\boldsymbol{x} \leftrightarrow \boldsymbol{y}$), and (S-14) is to control the target output $\boldsymbol{y}_{1:3}$ by P control, and (S-15) represents dosage limitations, and (S-16) represents contraindication of specific combinations of drugs, and (S-17) is the range for $\boldsymbol{x}$ to avoid physiologically unreasonable values. By solving this optimization problem by *Pyomo* [s2], the optimal drug feedforward input at $k = 0$ can be computed by $\boldsymbol{u}[0] = \Delta\boldsymbol{u}$.

In feedback control at $k \geq 1$ when variations are expected to be small around target operating points, *GCE* constraints (S-13) are replaced by

$$\Delta\boldsymbol{y}_{1:3} = \boldsymbol{G}_{1:3}\Delta\boldsymbol{x} \quad \text{(S-20)}$$

where $\boldsymbol{G}_{1:3} \in \mathbb{R}^{3\times6}$ is the submatrix of $\boldsymbol{G} \in \mathbb{R}^{5\times6}$ composed of its first three rows. By solving this optimization problem, the optimal drug input at k can be computed by $\boldsymbol{u}[k] = \boldsymbol{u}[k-1] + \Delta\boldsymbol{u}$.

Note that the in-silico validation studies indicated this controller's robustness against inter-patient variability in drug sensitivities and circulatory properties. [10] All the parameter values used in the controller is shown in S-II-C

S-II. Parameter Values

A. Drug Library's Parameters

The values of drug library are shown in TABLE S-1.

Constructing a comprehensive drug library would require extensive experiments, as drug responses exhibit both intra- and inter-subject variability. For this study, we developed a provisional drug library based on real data, sufficient to test whether the feedback component of the control system can compensate for disturbances despite imperfect quantization of pharmacologic effects. Evaluating the controller's robustness in vivo against such variability is a primary objective of this study.

All procedures were approved by the institutional ethics committee described in Section V-A2, and preliminary experiments were conducted on healthy canine models independent of those used in the main control studies.

To eliminate baroreflex influences, the Hering's nerve (a branch of the glossopharyngeal nerve) was transected. Cardiac output $CO(t)$ was measured continuously using a flow probe, and left atrial pressure P_{LA} was recorded via an indwelling catheter after thoracotomy. Each drug was then administered at half of its maximum dose (u_1: *DOB*, u_2: *NE*, u_3: *SNP*, u_4: *HES*) while hemodynamic responses $\boldsymbol{y}(t)$ were continuously monitored. Changes in $\boldsymbol{x}$ were analyzed using the method described in Section V-A3, and the resulting $\boldsymbol{B}$ matrix was obtained by averaging estimates from at least three animals. The gains of SBV changes by u_5: *PHL* were set to values deemed physiologically appropriate.

Samples with frequent arrhythmias or mean arterial pressure below 70 mmHg were excluded. For aggregated data, entries whose mean ± one standard deviation spanned zero were considered statistically insignificant and set to zero. Because the library was derived from animals without heart failure and after thoracotomy, it may not fully capture the pathophysiological conditions of heart failure models.

TABLE S-1. *Drug Library* Parameters in $\boldsymbol{B} = (b_{i,j})_{1\le i\le 6, 1\le j\le 5}$

Const.	Value	Unit	Description
b_{31}	0.606	(mmHg/ml)/(μg/kg/min)	*DOB* $\rightarrow E_{es}^{LV}$
b_{41}	0.168	(mmHg/ml)/(μg/kg/min)	*DOB* $\rightarrow E_{es}^{RV}$
b_{51}	6.341	(/min)/(μg/kg/min)	*DOB* $\rightarrow HR$
b_{61}	1.749$\cdot BW$	ml/(μg/kg/min)	*DOB* $\rightarrow SBV$
b_{12}	6.260	(mmHg·s/ml)/(μg/kg/min)	*NE* $\rightarrow R_s$
b_{22}	1.158	(mmHg·s/ml/(μg/kg/min)	*NE* $\rightarrow R_p$
b_{32}	9.234	(mmHg/ml)/(μg/kg/min)	*NE* $\rightarrow E_{es}^{LV}$
b_{42}	2.564	(mmHg/ml)/(μg/kg/min)	*NE* $\rightarrow E_{es}^{RV}$
b_{52}	37.18	(/min)/(μg/kg/min)	*NE* $\rightarrow HR$
b_{62}	8.029$\cdot BW$	ml/(μg/kg/min)	*NE* $\rightarrow SBV$
b_{13}	-0.283	(mmHg·s/ml)/(μg/kg/min)	*SNP* $\rightarrow R_s$
b_{23}	-0.052	(mmHg·s/ml)/(μg/kg/min)	*SNP* $\rightarrow R_p$
b_{33}	-0.211	(mmHg/ml)/(μg/kg/min)	*SNP* $\rightarrow E_{es}^{LV}$
b_{43}	-0.059	(mmHg/ml)/(μg/kg/min)	*SNP* $\rightarrow E_{es}^{RV}$
b_{63}	-1.891$\cdot BW$	ml/(μg/kg/min)	*SNP* $\rightarrow SBV$
b_{14}	-8$\times 10^{-4}$	(mmHg·s/ml)/ml	*HES* $\rightarrow R_s$
b_{64}	1.353	ml/ml	*HES* $\rightarrow SBV$
b_{65}	-1.000	ml/ml	*PHL* $\rightarrow SBV$
other $b_{i,j}$	0.000	N/A	everything else

B. Circulatory Equilibrium Framework's Parameters

The physical parameter values of Circulatory Equilibrium Framework in S-I-C are summarized in TABLE S-2. The ED-PVR's and ESPVR's constant parameters were provisionally estimated as follows:

1) For a canine heart failure model (AID = a), $\boldsymbol{y}$ was measured. The left-ventricular end-diastolic volume (V_{ed}^{LV}) was obtained via echocardiography, and the left-ventricular end-diastolic pressure (P_{ed}^{LV}) was approximated by $PAWP$.
2) The obtained V_{ed}^{LV} and P_{ed}^{LV} were applied to the single-point EDPVR estimation method [s1] for identifying EDPVR curve.
3) From this curve, 20 points were randomly sampled within $1.0 \le P_{ed}^{LV} \le 100$.
4) The inverse ESPVR estimation method [14] was then applied to estimate E_{es}^{LV}. In this method, EDPVR fitting to the sampled points are conducted, while simultaneously identifying EDPVR parameters (α_{ed}^{LV}, β_{ed}^{LV}, V_d^{LV}) and ESPVR parameter (V_0^{LV}) under constraints to avoid singularity.
5) For the right ventricle, the assumptions $E_{es}^{RV} = E_{es}^{LV}/4$, $V_d^{RV} = 0$, and $V_0^{RV} = 0$ were adopted. The measured $\boldsymbol{y}$ and estimated $\boldsymbol{x}$ were substituted into (S-5), and α_{ed}^{RV} and β_{ed}^{RV} were determined to satisfy the GCE constraint.

This procedure inevitably involves measurement errors, approximations, and assumptions. The aim of this study is not to establish precise parameter values, but to evaluate whether hemodynamic control can be achieved in vivo despite model discrepancies introduced by these parameters.

Our previous in-silico study [10] showed that control performance was preserved even with up to 30% deviations in physical parameters, including the constants of EDPVR and ESPVR.

TABLE S-2. *GCE* Model Parameters

Const.	Value	Unit	Description
W	0.129	min	*VRS* [15]
G_s, G_p	19.6, 3.49	ml/min/mmHg/kg	*VRS* [15]
α_{ed}^{LV}, α_{ed}^{RV}	0.10, 0.05	mmHg	EDPVR
β_{ed}^{LV}, β_{ed}^{RV}	0.13, 0.15	/ml	EDPVR
V_d^{LV}, V_d^{RV}	7.80, 0	ml	EDPVR
V_0^{LV}, V_0^{RV}	7.75, 0	ml	ESPVR

TABLE S-3. Controller Parameters

Param.	Value	Description
$y_{1d} = MAP_d$	85	desired MAP
$y_{2d} = CI_d$	4.5	desired CI
$y_{3d} = P_{LA\_d}$	10	desired P_{LA}
$\overline{u}_1, \cdots, \overline{u}_5$	10, 0.3, 10, 250, 250	max values
$\underline{\boldsymbol{x}}$	$[0.1, 0.003, 0.2, 0.02, 50, 100]^T$	lower limit for $\boldsymbol{x}$
$\overline{\boldsymbol{x}}$	$[10.0, 2.5, 8.0, 5.0, 220, 800]^T$	upper limit for $\boldsymbol{x}$
$\boldsymbol{W}$	$\boldsymbol{W}_u^{-T}\boldsymbol{I}_5\boldsymbol{W}_u^{-1} \in \mathbb{R}^{5\times 5}$	weight to drug use
$\boldsymbol{W}_u$	diag($[\overline{u}_1, \cdots, \overline{u}_5]$)	input scaler
S	{(2,3), (4,5)}	contraindication
$\boldsymbol{K}_p$	0.5 $\boldsymbol{I}_3$	P gain in (S-19)

TABLE S-4. Measured CI via Thermodilution

EID	$k=0$	$k=1$	$k=2$	$k=3$	$k=4$	$k=5$
exp1	3.58±0.18	3.11±0.09	3.92±0.09	3.85±0.16	4.06±0.00	3.99±0.09
exp2	3.47±0.09	2.43±0.09	3.75±0.00	4.86±0.25	4.30±0.19	3.96±0.16
exp3	2.99±0.19	3.26±0.09	4.17±0.00	4.23±0.25	5.00±0.28	4.72±0.09
exp4	4.86±0.19	4.37±0.16	3.05±0.09	4.51±0.09	4.65±0.25	4.30±0.19
exp5	3.05±0.09	5.55±0.19	2.92±0.00	4.93±0.09	4.79±0.00	3.89±0.09

C. Controller's Parameters

The parameters for the controller design in S-I-D are shown in TABLE S-3.

S-III. Experimental Data of $CI[k]$ and $P_{LA}[k]$

Measurements of CI obtained by thermodilution and P_{LA} estimated from $PAWP$ required manual procedures and therefore were subject to measurement variability.

Following the procedure described in Section V-A5, $PAWP$ and thermodilution measurements were repeated multiple times at each discrete step. The resulting mean and standard deviation are reported to characterize the variability associated with these measurements.

TABLE S-4 summarizes the experimental data of $CI[k]$. It was calculated from the measured CO and the BSA of each animal. Values are presented as mean ± standard deviation ($N = 3$ measurements). Across all experiments and discrete steps, the mean within-step standard deviation was 0.125 L/min/m^2, corresponding to 2.8% of the target value ($CI_d = 4.5$ L/min/m^2). Across all experiments and discrete steps, the mean standard deviation among the three thermodilution measurements was 0.125 L/min/m^2, corresponding to 2.8% of the target value ($CI_d = 4.5$ L/min/m^2).

TABLE S-5 summarizes the experimental data of $P_{LA}[k]$ estimated from $PAWP$. At each discrete step, $PAWP$ was measured before and after the thermodilution procedure, and the resulting mean and standard deviation are reported ($N = 2$

TABLE S-5. Measured P_{LA} via $PAWP$

EID	$k=0$	$k=1$	$k=2$	$k=3$	$k=4$	$k=5$
exp1	17.9±0.1	12.2±0.6	13.5±0.0	10.9±0.6	10.8±0.3	9.9±0.1
exp2	22.5±0.5	7.7±0.1	11.4±0.1	10.2±0.1	9.0±0.1	9.4±0.1
exp3	14.7±1.0	7.3±0.1	9.4±0.0	9.7±0.2	10.2±0.7	9.1±0.4
exp4	21.1±0.2	9.6±0.2	9.5±1.0	10.1±0.4	8.9±0.9	8.9±0.1
exp5	17.5±0.5	11.4±0.9	9.8±0.0	9.8±0.2	9.2±0.1	8.8±0.2

TABLE S-6. LVEF: Before and After HF Induction

AID	LVEF Before	LVEF After
a	60.1%	39.0%
b	60.5%	33.6%
c	62.4%	32.6%
d	53.0%	30.8%
Mean±SD	$59.0 \pm 4.1\%$	$34.0 \pm 3.5\%$

measurements). Across all experiments and discrete steps, the mean standard deviation was 0.32 mmHg, corresponding to 3.2% of the target value ($P_{LA\_d} = 10$ mmHg).

These statistics are provided to characterize measurement variability in the present experiments. Because of the limited sample size, they should be interpreted as descriptive observations rather than formal estimates of measurement precision.

S-IV. Discrete *CV parameters* $\boldsymbol{x}$ Estimation

Given the measured hemodynamics $\boldsymbol{y}$ in clinical practice, *CV parameters* $\boldsymbol{x}[k]$ can be estimated as follows. Here, t represents continuous time, and k represents the discrete step for hemodynamic measurements after the convergence of drug effects.

First, x_1: $R_s[k]$ and x_2: $R_p[k]$ can be discretely computed by basic vascular resistance model as

$$R_s[k] = \frac{60\,(MAP(t) - P_{RA}(t))}{CO[k]} \tag{S-21}$$

$$R_p[k] = \frac{60\,(PAP(t) - P_{LA}[k])}{CO[k]}. \tag{S-22}$$

Next, x_3: $E_{es}^{LV}[k]$ and x_4: $E_{es}^{RV}[k]$ can be estimated using our techniques [14]. The key formula for estimatitng E_{es}^{LV} is the inverse function of the *Frank-Starling principle* as

$$E_{es}^{LV}[k] = \frac{x_5\left(y_4 + \dfrac{x_1}{60}y_2\right)}{\dfrac{x_5}{\beta_{ed}^{LV}}\,g_l(y_3) - y_2} \text{ where } \frac{x_5}{\beta_{ed}^{LV}}\,g_l(y_3) \neq y_2 \tag{S-23}$$

$$E_{es}^{RV}[k] = \frac{x_5\left(y_3 + \dfrac{x_2}{60}y_2\right)}{\dfrac{x_5}{\beta_{ed}^{RV}}\,g_r(y_4) - y_2} \text{ where } \frac{x_5}{\beta_{ed}^{RV}}\,g_r(y_4) \neq y_2. \tag{S-24}$$

Note that $g_l(y_3)$ and $g_r(y_4)$ are eqivalent to (S-7) and (S-8). Because this method depends on the discrete measurement of y_2: $CO[k]$, x_3: $E_{es}^{LV}[k]$ and x_4: $E_{es}^{RV}[k]$ can be also estimated discretely. We recognize that the estimation of E_{es} has been a challenging task, and that this estimation method may remain imperfect. In particular, while it is mathematically reasonable to extend a method validated for the left heart to the right heart, such an extension would likely require meticulous additional experiments. In this control study, we assume that this provisional estimation method may contribute to measurement errors, and our objective is to evaluate whether the control system can function robustly despite these errors.

Finally, x_6: $SBV[k]$ can be discretely estimated by [15] as

$$SBV[k] = W\left\{CO + BW(G_s P_{RA}(t) + G_p P_{LA}[k])\right\} \tag{S-25}$$

where W, G_s, G_p are (*VRS*) parameters in min, ml/min/mmHg/kg, and ml/min/mmHg/kg respectively, and BW is body weight in kg. Since this depends on y_2: $CO[k]$, y_3: $P_{LA}[k]$, and y_4: $P_{RA}(t)$, x_6: SBV is also estimated discretely.

S-V. Visualization Tool for Hemodynamic Control

A visualization tool for hemodynamic control system was developed (Fig. S-1) that integrated hemodynamic measurements $\boldsymbol{y}[k]$ from multiple monitoring devices, estimated states $\boldsymbol{x}[k]$, control inputs $\boldsymbol{u}[k]$, and predicted values of $\boldsymbol{x}[k+1]$ and $\boldsymbol{y}[k+1]$.

First, this tool allowed intuitive monitoring of the hemodynamic control process and drug delivery progression. It visualizes the relationship between P_{LA} and CI based on the *Forrester Classification*, facilitating clinical interpretation.

Second, the gap between predicted and measured values increased transparency of cardiovascular causality and debuggability. For example, transient HR decrease from $k=0$ to $k=1$ occurred opposite to the predicted trend, indicating a notable model–plant discrepancy, likely due to the *Bezold–Jarisch reflex* [17]. Similarly, E_{es}^{LV} and E_{es}^{RV} varied contrary to predictions, potentially reflecting coronary hypoperfusion. In contrast, R_s, R_p, and SBV converged approximately to intended values. These results demonstrate that model predictive control, explicitly incorporating hemodynamic causal structure, enables analysis of transient deviations and insight into underlying physiology, enhancing system transparency and debuggability.

S-VI. Left Ventricular Ejection Fraction ($LVEF$) Before and After HF Induction

The changes in $LVEF$ before and after HF induction are summarized in TABLE S-6 for the four canine models used in this study.

References

[s1] S. Klotz, and et al., "Single-beat estimation of end-diastolic pressure-volume relationship: a novel method with potential for noninvasive application," *American Journal of Physiology-Heart and Circulatory Physiology*, vol. 291, no. 1, pp. H403–H412, 2006.

[s2] M. L. Bynum and et al., "Pyomo–optimization modeling in python," *Springer Science & Business Media*, Third Edition, vol. 67, 2021.

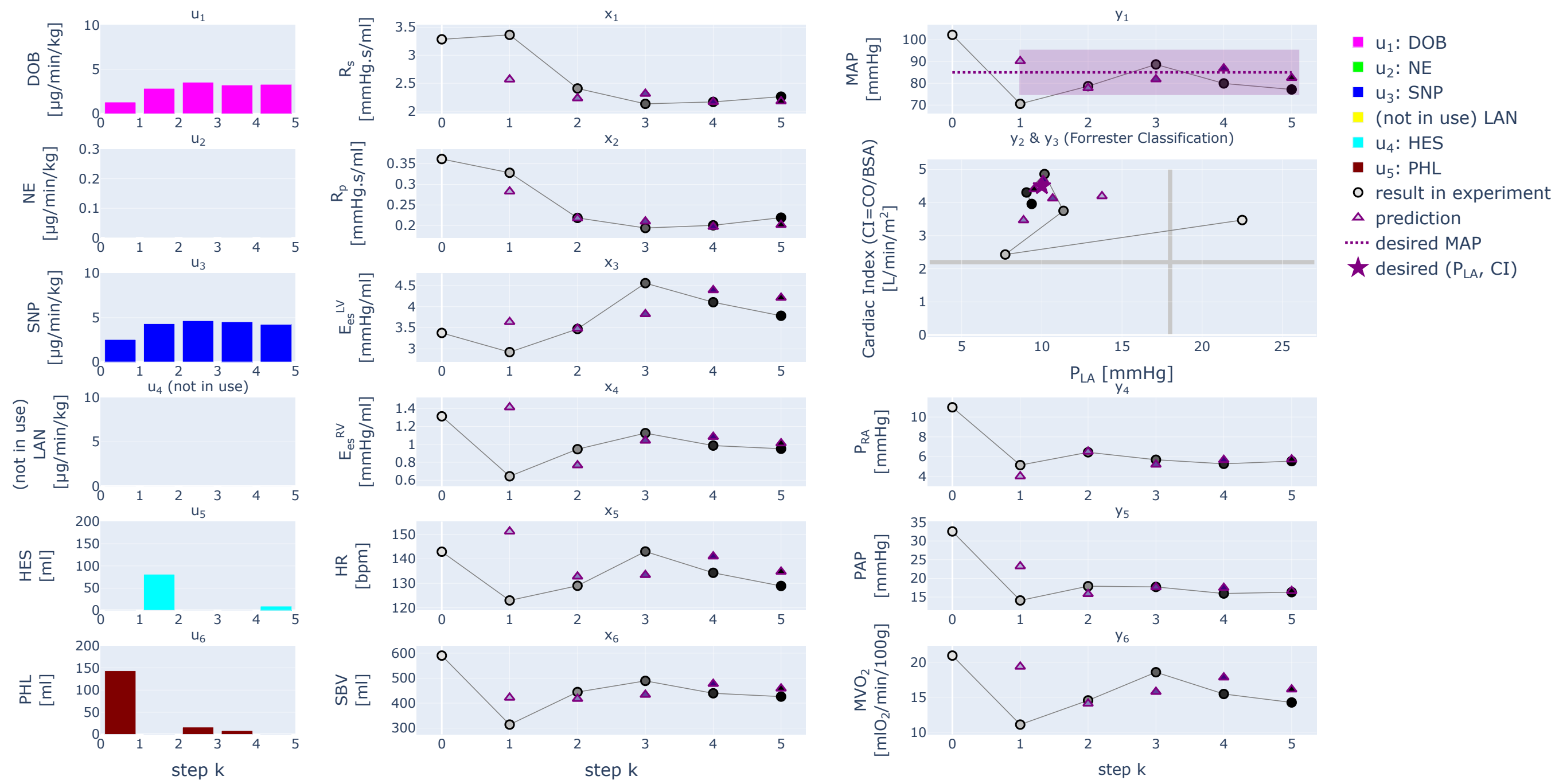

Fig. S-1. Visualization Tool of Hemodynamics Predictions and Outcomes; △: Predicted values of $\boldsymbol{y}[k]$ and $\boldsymbol{x}[k]$ based on inputs applied between $u[k-1]$ and $u[k]$ using the drug infusion model and *GCE*. ◯: Results or estimated values based on observed measurements.
